# Safety and Efficacy of Leadless Pacemakers: A New Era of Pacing

**DOI:** 10.19102/icrm.2018.090701

**Published:** 2018-07-15

**Authors:** Zaid Ammari, Mubbasher Syed, Mohammad Al-Sarie, Saima Karim, Blair Grubb

**Affiliations:** ^1^Department of Internal Medicine, University of Toledo, Toledo, OH, USA; ^2^Department of Cardiovascular Medicine, University of Toledo, Toledo, OH, USA

**Keywords:** Leadless, leadless pacemaker, pacing

## Abstract

Leadless pacemakers are evolving as a new technologic alternative to conventional transvenous pacemakers, though potential short-term and long-term complications have been recognized. The two currently available right ventricular leadless pacing systems are the Nanostim™ Leadless Cardiac Pacemaker (Abbott Laboratories, Chicago, IL, USA) and the Micra™ Transcatheter Pacing System (Medtronic, Minneapolis, MN, USA). This review aims to highlight the safety and efficacy of leadless pacemakers using these two devices as exemplars.

## Introduction

Since the first pacing device was implanted almost 60 years ago, there has been a recent and consistent rise in the use of permanent pacemakers for conduction system defects, chronotropic incompetence, symptomatic bradycardia, and sick sinus syndrome.^1–4^ Nearly one million pacemaker implants are performed annually, with more than one-quarter completed in the United States.^5^ At the time of its introduction, innovators foresaw an ever-increasing demand for a device with the ability to assist with the electrical conduction of the heart. They have since been trying to improve the pacemaker’s design and reduce complications associated with this piece of equipment.

It has long been recognized that most adverse outcomes related to the implantation of a conventional pacemaker are attributed to the pocket within which it lies or the leads of the device extending into the endocardium. Such outcomes include, but are not limited to, hematoma of the pocket, pocket infection, lead infection, endocarditis, lead fracture, and lead dislodgement. The simplest solution for many of these problems was deemed to be to eliminate the leads and the need for a pocket. As early as 1970, conceptual designs were presented that detailed the idea of miniaturizing a pacing device and implanting it directly onto the endocardium, but there were more than a few limitations to the concept.^6,7^

Since then, in the last several decades, technological advancements have allowed us to imbue pacing devices with longer battery life, develop smaller prototypes, and introduce more reliable delivery systems. Meanwhile, the ability to pace the human heart without creating an artificial pocket or employing endovascular active or passive fixation leads has been revisited by St. Jude Medical (St. Paul, Minnesota, MN, USA)—now part of Abbott Laboratories (Chicago, IL, USA)—and Medtronic (Minneapolis, MN, USA). These companies’ efforts will be discussed henceforth.

## Transvenous pacemakers and related complications

In the initial era of pacemaker implantation, there was a high proportion of device-related complications, which has since declined over the decades. This reduction in adverse outcomes can be attributed to better device design, technological advancements in implantation techniques and apparatuses, and growth of operator expertise in the field. That said, there is still much to be achieved in the face of a reported complication rate of 8% to 10% with transvenous pacemaker implantation.^8^

The most common complications associated with traditional pacemakers during intraoperative maneuvering and include pneumothorax, cardiac tamponade, and pocket hematomas; these can be as frequent as 5.1%.^9,10^ Complications can also be related with extension of the leads through the venous space, leading to fracturing of the leads, loss of insulation, obstruction, thrombosis, and tricuspid regurgitation.^11,12^ These leads can also serve as a nidus for infection, which can extend down to the endocardial surface. Endocarditis related with transvenous leads has been associated with an up to 31% mortality risk in some reports.^13,14^ It has been well-established^15^ that even single-chamber pacemakers, which reportedly demonstrate a lower rate of complications than do dual-chamber devices, require surgical reintervention in more than 2% of all device implant cases. The majority of these revisions are related in some fashion to the transvenous leads.^15^

In addition, the subcutaneous pocket created for the placement of the device can lead to skin erosion, localized infection, and bacteremia. Such pocket-related complications are reported in 1% to 2% of all procedures performed.^16,17^ Sometimes, patients return with lead noise related to the insulation of the conductors involved. These individuals invariably undergo reintervention, which itself has an increased risk for complications.^18,19^ It is not uncommon for patients with lead-related complications to require lead extraction procedures, which are often performed using mechanical snares or laser technology and can be associated with injury to the vessels and/or endocardium, with significant associated morbidity.^20,21^

## Leadless pacemakers

### Leadless cardiac pacing systems

The two available right ventricular leadless pacing systems are the Nanostim™ Leadless Cardiac Pacemaker (Nanostim™ LCP; Abbott Laboratories, Chicago, IL, USA) and the Micra™ Transcatheter Pacing System (Micra™ TPS; Medtronic, Minneapolis, MN, USA). The characteristics of both of these systems are summarized in **Table 1**.

The Nanostim™ LCP (Abbott Laboratories, Chicago, IL, USA) was developed by St. Jude Medical (St. Paul, MN, USA). It measures 41.4 mm in length × 5.99 mm in width and weighs 2 g. The device comprises a lithium carbon monofluoride battery with sensing and pacing electrodes enclosed in a 1 cm^3^ capsule. VVI/R mode is used for pacing, rate response is provided by a temperature-based sensor, and 250-kHz electrocardiogram surface electrodes using the Merlin™ Patient Care System Model 3650 (Abbott Laboratories, Chicago, IL, USA) are used for device interrogation and programming. Battery longevity is estimated to range from 9.8 years to 14.7 years based on pacing burden with 100% pacing at 2.5 V at 0.4 ms/60 bpm and 1.5 V at 0.25 ms/60 bpm, respectively. The Nanostim™ LCP (Abbott Laboratories, Chicago, IL, USA) is designed to be retrievable.^22–27^

In comparison, the Micra™ TPS (Medtronic, Minneapolis, MN, USA) measures 25.9 mm in length × 6.67 mm in width and weighs 2 g. The device comprises a lithium silver vanadium oxide/carbon monofluoride battery with sensing and pacing electrodes enclosed in a 0.8 cm^3^ capsule. VVI/R mode is used for pacing, rate response is provided by an accelerometer, and radiofrequency current using the CareLink™ 2090 Programmer (Medtronic, Minneapolis, MN, USA) is used for device interrogation and programming. Battery longevity is estimated to range from 4.7 years to 10 years, based on pacing burden with 100% pacing at 2.5 V at 0.4 ms/60 bpm and 1.5 V at 0.24/60 bpm, respectively. The Micra™ TPS (Medtronic, Minneapolis, MN, USA) is not designed to be retrievable after implantation.^22–27^

### Implantation technique/procedure

By definition, a leadless pacemaker is a self-contained, encapsulated device that includes surface sensing and pacing electrodes and a generator. It is implanted percutaneously in the right ventricle via a femoral vein sheath. The Micra™ TPS (Medtronic, Minneapolis, MN, USA) is wider than the Nanostim™ LCP (Abbott Laboratories, Chicago, IL, USA); a larger 23-French introducer sheath is typically used for the former versus a smaller 18-French introducer sheath for the latter. The implant procedure is performed in the electrophysiology laboratory under local anesthetic and conscious sedation in a manner similar to that used for transvenous device implantation. Under fluoroscopic guidance, the leadless pacemaker is advanced from the inferior vena cava to the right atrium and through the tricuspid valve into the right ventricle using a deflectable system. It is implanted into the apicoseptal area of the right ventricle endocardium. In the Micra™ TPS (Medtronic, Minneapolis, MN, USA), fixation is achieved using a linear deployment system with self-expanding nitinol tines. The Nanostim™ LCP (Abbott Laboratories, Chicago, IL, USA) employs an active fixation mechanism with a rotational screw-in helix, in combination with tines. After fixation, the pacemaker is released from the deflectable system. However, prior to releasing the leadless pacemaker from the tethers, pacing threshold, sensing amplitude, and impedance should be checked and a tug test performed under fluoroscopic guidance to ensure stability of the device.^22,27–29^

### Available studies on efficacy and safety

The LEADLESS trial, a prospective nonrandomized multicenter study, was the first study of leadless pacemaker technology performed using human subjects.^30^ It was conducted between December 2012 and April 2013 and enrolled 33 patients from three European centers. All patients underwent Nanostim™ LCP (Abbott Laboratories, Chicago, IL, USA) implantation and were followed up via serial assessments over a period of 12 weeks. The primary endpoint of the study was freedom from complications. Implant success rate was 97% with a mean procedure time of 28 minutes ± 17 minutes and hospital discharge occurring within 31 hours ± 20 hours of implantation. At 12 weeks’ follow-up, 94% of patients were complication-free, with one patient developing cardiac tamponade during implantation. Mean R-wave amplitude, pacing threshold, and impedance were used to assess performance, with significant improvement in all three measures at 12 weeks postoperation versus at implantation. The 31 patients who were complication-free at 12 weeks were subsequently enrolled in a follow-up study.^31^ In a mean follow-up of 1.2 years ± 0.1 years, no complications related to the leadless pacemakers were reported. Performance measures remained stable throughout the study duration, with an adequate rate response occurring in all study personnel.

A second, larger, prospective nonrandomized multicenter study, LEADLESS II, involved 526 patients from 56 centers in the United States, Canada, and Australia.^32^ All patients underwent Nanostim™ LCP (Abbott Laboratories, Chicago, IL, USA) implantation with an implant success rate of 96% and mean procedure time of 28.6 minutes ± 17.8 minutes. Of the total patient cohort, 6.5% experienced device-related serious adverse events, with 40 events experienced by 34 patients. Cardiac perforation, vascular complications, and arrhythmias during device implantation occurred in 1.6%, 1.2%, and 0.6% of the total cohort, respectively. Two patients (0.4%) experienced device migration during implantation and six patients (1.1%) experienced device dislodgement, four to the pulmonary artery and two to the right femoral vein. Four patients (0.8%) required device retrieval secondary to elevated pacing threshold. Other device-related serious adverse events included hemothorax, autonomic dysfunction, cerebrovascular accidents, and contrast-induced nephropathy. Device-related serious adverse events in relation to operator experience were 6.8% for the first 10 devices implanted versus 3.6% for subsequent implants. Performance measures, including mean sensing and pacing threshold values, improved significantly from the time of implantation to at one year after surgery, with a statistically significant increase in mean R-wave amplitude and a decrease in mean pacing capture threshold. Pacing function was further assessed by a 24-hour ambulatory Holter monitor in 30 patients. At one-year follow-up, 50.3% ± 39.9% of patients had ventricular pacing, with a mean heart rate of 71.2 bpm ± 9.8 bpm. There were no reported pauses lasting more than 2.0 seconds, undersensing, or a failure to capture episodes. Oversensing of T-waves was reported in four patients; all were asymptomatic with no reported adverse events.

The Nanostim™ LCP (Abbott Laboratories, Chicago, IL, USA) has yet to be approved by the United States Food and Drug Administration (US FDA), as battery advisories have put a key study on hold, with reported battery malfunctions occurring in 0.5% of the patient cohort at between 29 and 37 months after device implantation.^29^

The safety and efficacy of the Micra™ TPS (Medtronic, Minneapolis, MN, USA) was evaluated by a prospective multicenter study involving 744 patients from 56 centers in 19 countries who met class I or II guidelines for single-chamber pacing. Device implantation was attempted in 725 patients, with an implant success rate of 99.2% and mean procedure time of 23.0 minutes ± 15.3 minutes. Safety was evaluated during serial follow-up appointments and compared with published data on conventional pacemakers at six months postimplantation. Major complications were experienced by 4.0% of the patients, with a total of 28 events occurring in 25 patients. Traumatic cardiac injury, vascular complications in the groin, and venous thromboembolism occurred in 1.6%, 0.7%, and 0.3% of the patients, respectively. There were no reported device dislodgements or migrations, though reported events did include elevated pacing threshold, heart failure, acute myocardial infarction, and autonomic dysfunction, with one reported death occurring that was believed to be secondary to metabolic acidosis. When compared with the published data on conventional pacemakers (control patients), after adjustment for baseline characteristics, the study participants had fewer complications than did the control group at six months postimplantation, with a hazard ratio of 0.46 (95% confidence interval: 0.28–0.74).^33,34^ A longer-term study found similar safety results at 12 months after surgery, along with good electrical performance for up to 24 months postimplantation.^35^

With respect to the efficacy of the Micra™ TPS (Medtronic, Minneapolis, MN, USA), the primary efficacy endpoint in the same study was a low pacing capture threshold (≤ 2 V), with a stable increase ≤ 1.5 V at six months postimplantation. Efficacy analysis revealed stable electrical performance, including a mean pacing capture threshold at a pulse width of 0.24 ms, mean R-wave amplitude, and impedance.^34^

Da Costa et al. evaluated the safety, efficacy, and feasibility of Micra™ TPS (Medtronic, Minneapolis, MN, USA) implantation in 14 patients with difficult access or contraindications for conventional pacemaker placement.^36^ Implantation was successful in 100% of patients, with a mean procedure time of 45 minutes ± 7 minutes. No direct access site complications, device dislodgements, or cases of embolization were reported. One patient experienced ventricular fibrillation arrest one day after pacemaker implantation, with normal unchanged parameters present on pacemaker testing after the restoration of normal sinus rhythm by cardioversion. Mean capture threshold, sensed R-waves, and impedance were stable at three months after surgery. This study highlighted the safety and efficacy of leadless pacemakers in patients with venous abnormalities and kidney failure with limited venous access.^36^

In a case series including six patients who underwent Micra™ TPS (Medtronic, Minneapolis, MN, USA) placement, implantation was successful in all patients. As compared with at immediately following implantation, sensing and pacing values were stable at one month and six months after implantation.^37^

In a systematic review comparing the incidence of lead perforation in conventional versus leadless pacemaker implantation, data from more than 60,000 patients undergoing conventional pacemaker implantation demonstrated a low incidence of lead perforation (< 1%), whereas there was a 1.5% incidence of cardiac perforation in the two included leadless pacemaker studies. The difference in the incidence of lead perforation is attributable to operator experience with this new technology and differences between the designs of the leadless pacemaker devices.^38^

St. Jude previously reported the mean estimated battery life of a leadless cardiac pacemaker to be 15 years in patients implanted for six months.^32^ The Micra TPS™ (Medtronic, Minneapolis, MN, USA) uses autocapture algorithms to improve its battery life. However, this usage raises the question of whether the algorithm for autocapture provides adequate safety. A study involving patients implanted with the Micra™ TPS (Medtronic, Minneapolis, MN, USA) found at six months that the estimated battery life was 12.5 years.^33^

### Leadless pacemaker retrieval

Data on end-of-life management of leadless pacemakers remain controversial, with options including placement of a new device adjacent to the old one or retrieval of the old leadless pacemaker and implantation of a new one.^39^ In a study involving patients enrolled in three centers with Nanostim™ LCP (Abbott Laboratories, Chicago, IL, USA) devices implanted, 16 patients underwent device retrieval attempts. A total success rate of 94% was reported: there was a 100% success rate in patients with leadless pacemakers implanted for less than six weeks and a 91% success rate in patients with leadless pacemakers implanted for more than six weeks. There were no reported postprocedural adverse events at 30 days’ follow-up after retrieval.^40^ Separately, Lakkireddy et al. evaluated 73 patients for Nanostim™ LCP (Abbott Laboratories, Chicago, IL, USA) retrieval with a success rate of 90.4%.^41^ Serious complications of device retrieval included the development of atriovenous fistula and migration to the pulmonary artery. In the same study, 115 patients received an additional leadless pacemaker or conventional transvenous pacemaker adjacent to the abandoned Nanostim™ LCP device (Abbott Laboratories, Chicago, IL, USA), with no adverse events reported.^41^

In 13 patients implanted with the Micra™ TPS (Medtronic, Minneapolis, MN, USA) who required device revision, retrieval was attempted in 10 and a success rate of 80% was achieved.^42^ Several case reports have indicated successful device retrieval attempts were made at various stages after implantation for reasons including infection and elevated capture thresholds.^43,44^ Giocondo reported a case of unsuccessful retrieval of a Micra™ TPS (Medtronic, Minneapolis, MN, USA).^45^

## Left ventricular pacing

It is worth noting that, in addition to the aforementioned two right ventricular pacing systems, a single left ventricular (LV) pacing option, the WiCS^®^-LV pacing system (EBR Systems, Sunnyvale, CA, USA), is also under development, though it is not yet US FDA-approved. The system provides leadless pacing by converting ultrasound energy to electrical energy.^46^ One study, the Safety and Performance of Electrodes Implanted in the Left Ventricle (SELECT-LV) trial,^47^ included 35 patients indicated for CRT who had either failed conventional CRT or who required a device upgrade but were deemed unsuitable for conventional CRT; these individuals were implanted with an LV endocardial pacing electrode and a subcutaneous pulse generator. Results indicated the primary performance endpoint (biventricular pacing on 12-lead electrocardiogram at one month) was achieved in 33 (97.1%) of the 34 patients, while 28 patients (84.8%) demonstrated improvement in the clinical composite score at six months and 21 (66%) patients showed a positive echocardiographic CRT response (≥ 5% absolute increase in LV ejection fraction). No cases of pericardial effusion were found, but serious procedure/device-related events occurred in three patients (8.6%) within 24 hours of surgery and in eight patients (22.9%) between 24 hours and one month after surgery.

In addition, two studies employing the Wireless Stimulation Endocardially for Cardiac Resynchronization Therapy (WiSE-CRT) trial’s patient cohort^46,48^ have been published. The first, an initial first-in-man implantation study,^46^ included three patients: one with an existing implantable cardioverter-defibrillator, one with a CRT system whose LV lead did not capture, and one who was a CRT nonresponder. At the time of the study, all three patients were reported to have been successfully treated via WiCS^®^-LV pacing system implantation (EBR Systems, Sunnyvale, CA, USA). Acute electrical pacing thresholds postimplantation ranged from 0.7 V to 1.0 V at 0.5 ms and all patients retained capture at six months. Additionally, New York Heart Association (NYHA) functional class significantly changed, from III in two patients and IV in one patient to I in one patient, II in one patient, and II/III in one patient, respectively. Furthermore, LV ejection fraction increased from 23.7% ± 3.4% to 39% ± 6.2% (p < 0.017). The second published study^48^ considered all 17 patients from the WiSE-CRT trial, reporting that system implantation was achieved in 13 of the 17 patients (76.5%) and that mean R-wave amplitude was 5.6 mV ± 3.2 mV and mean pacing threshold was 1.6 V ± 1.0 V, respectively. Of those successfully implanted, biventricular pacing was recorded in 83% and 92% of the patients at one month and six months, respectively. At the six-month follow-up, two-thirds of the patients had at least one NYHA functional class change, and LV ejection fraction was also significantly increased (p < 0.01) by six points. No sufficient pacing thresholds were found in one patient and pericardial effusion occurred in three patients. Though these studies represent a start, further research using larger populations is required to better elucidate the safety and efficacy of the WiCS^®^-LV pacing system (EBR Systems, Sunnyvale, CA, USA) and other potential LV pacing options.

## Conclusion

Initial data on leadless pacemaker implantations are promising in terms of decreased adverse effects related with transvenous lead and pocket infections. Randomized multicenter clinical trials are warranted to determine the efficacy and safety of leadless pacemakers in comparison with conventional transvenous pacemakers.

## Figures and Tables

**Table 1:** Available Right Ventricular Leadless Cardiac Pacemaker^22–27^

	Nanostim™ LCP	Micra™ TPS
Manufacturer	Initially St. Jude Medical (St. Paul, MN, USA), now Abbott Laboratories (Chicago, IL, USA)	Medtronic (Minneapolis, MN, USA)
Dimensions	41.4 mm × 5.99 mm	25.9 mm × 6.67 mm
Volume	1 cm^3^	0.8 cm^3^
Weight	2 g	2 g
Chamber paced	Right ventricle	Right ventricle
Pacing	VVI/R mode	VVI/R mode
Sensing	Temperature-based	Accelerometer
Interrogation and programming	Merlin™ Patient Care System Model 3650 (Abbott Laboratories, Chicago, IL, USA)	Medtronic CareLink™ 2090 Programmer (Medtronic, Minneapolis, MN, USA)
Telemetry	Conductive	Radiofrequency
Battery	Lithium carbon monofluoride	Lithium silver vanadium oxide/carbon monofluoride
Battery longevity	9.8–14.7 years*	4.7–10 years*

LCP: Leadless Cardiac Pacemaker; TPS: Transcatheter Pacing System.

*Based on pacing burden with 100% pacing at 2.5 V at 0.4 ms/60 bpm and 1.5 V at 0.24 ms/60 bpm.
